# WISDOM-II: Screening against multiple targets implicated in malaria using computational grid infrastructures

**DOI:** 10.1186/1475-2875-8-88

**Published:** 2009-05-01

**Authors:** Vinod Kasam, Jean Salzemann, Marli Botha, Ana Dacosta, Gianluca Degliesposti, Raul Isea, Doman Kim, Astrid Maass, Colin Kenyon, Giulio Rastelli, Martin Hofmann-Apitius, Vincent Breton

**Affiliations:** 1 Department of Bioinformatics, Fraunhofer Institute for Algorithms and Scientific Computing (SCAI), 53754 Sankt Augustin, Germany; 2 Dipartimento di Scienze Farmaceutiche, Università di Modena e Reggio Emilia. Via Campi 183, 41100 Modena Italy; 3 LPC Clermont-Ferrand, Campus des Cézeaux, 63177 Aubière Cedex, France; 4 CSIR Biosciences, Modderfontein, Johannesburg, 1645, South Africa; 5 School of Biological Sciences and Technology, Chonnam National University, Gwang-Ju, South Korea; 6 Fundacion IDEA, Centro de Biociencias, Hoyo de la Puerta, Baruta 1080, Venezuela

## Abstract

**Background:**

Despite continuous efforts of the international community to reduce the impact of malaria on developing countries, no significant progress has been made in the recent years and the discovery of new drugs is more than ever needed. Out of the many proteins involved in the metabolic activities of the *Plasmodium *parasite, some are promising targets to carry out rational drug discovery.

**Motivation:**

Recent years have witnessed the emergence of grids, which are highly distributed computing infrastructures particularly well fitted for embarrassingly parallel computations like docking. In 2005, a first attempt at using grids for large-scale virtual screening focused on plasmepsins and ended up in the identification of previously unknown scaffolds, which were confirmed in vitro to be active plasmepsin inhibitors. Following this success, a second deployment took place in the fall of 2006 focussing on one well known target, dihydrofolate reductase (DHFR), and on a new promising one, glutathione-S-transferase.

**Methods:**

In silico drug design, especially vHTS is a widely and well-accepted technology in lead identification and lead optimization. This approach, therefore builds, upon the progress made in computational chemistry to achieve more accurate *in silico *docking and in information technology to design and operate large scale grid infrastructures.

**Results:**

On the computational side, a sustained infrastructure has been developed: docking at large scale, using different strategies in result analysis, storing of the results on the fly into MySQL databases and application of molecular dynamics refinement are MM-PBSA and MM-GBSA rescoring. The modeling results obtained are very promising. Based on the modeling results, *In vitro *results are underway for all the targets against which screening is performed.

**Conclusion:**

The current paper describes the rational drug discovery activity at large scale, especially molecular docking using FlexX software on computational grids in finding hits against three different targets (PfGST, PfDHFR, PvDHFR (wild type and mutant forms) implicated in malaria. Grid-enabled virtual screening approach is proposed to produce focus compound libraries for other biological targets relevant to fight the infectious diseases of the developing world.

## Background

Discovering hits with the potential to become usable drugs is a critical first step to ensure a sustainable global pipeline for innovative anti-malarial products. While the establishment of public-private partnerships has helped to stimulate product R&D for some neglected diseases, increased emphasis needs to be placed on the high-risk early discovery phase.

This paper describes an established hit discovery strategy for neglected diseases through *in silico *screening using computing grid infrastructures, as a very cost effective way to select the most promising drug-like molecules to address Plasmodium multi-drug resistance. Here the aim is to counter-act malaria by finding hits to multiple targets. This is so far the first large scale *in silico *drug finding initiative against malaria and neglected diseases.

The project fits in the drug discovery pipeline between initiatives like the TDR drug target portfolio programme [1], which aims at developing a prioritized drug target portfolio, and initiatives like DNDi [2], which address pre-clinical research on new lead compounds. WISDOM project enables the cost effective selection of focused compound libraries for drug targets to allow cheap and small scale *in vitro *and *in vivo *tests affordable by all research laboratories, even in less developed countries.

This approach builds upon the progress made in computational chemistry to achieve more accurate *in silico *docking and in information technology to design and operate large-scale grid infrastructures.

This paper describes the collaborative framework, which has been established between bio-informaticians, biochemists, pharmaceutical chemists, biologists and grid experts, in order to produce and make selected lists of potential inhibitors available. It also aims at publicizing the service for research laboratories interested to use it for their own preferred target.

### WISDOM, a virtual docking service on grids

Due to very high costs associated to the drug discovery process as well as due to late stage attrition rates, novel and cost effective strategies are absolutely needed for combating the neglected diseases, like malaria. Virtual high throughput screening is a technique, which can screen millions of compounds rapidly, reliably and cost effectively on a computer [3,4].

There are millions of chemical compounds available in the labs and also in 2D, 3D electronic databases due to advances in the combinatorial chemistry, but it is nearly impracticable to synthesize them [5]. Moreover it is labour-intensive and very expensive to screen such a high number of compounds in experimental labs by high throughput screening (HTS). Besides the heavy costs (required for developing efficient and reliable assays) the hit rate in HTS is quite low [5].

In addition to the availability of a huge number of chemical compounds, there is also a significant increase in the number of resolved X-ray crystal structures, most of which are freely available from the Brookhaven protein database [6].

The presence of sound open source electronic data of chemical compounds as well as the data for macromolecules like proteins, enzymes facilitated virtual high throughput screening (vHTS). Virtual high throughput screening (vHTS) by molecular docking serves as a complementary or alternative technique to experimental high throughput screening (HTS). *In silico *drug design, especially high throughput virtual screening is a widely-used and well-accepted technology in new lead identification and optimization [7,8].

The downside to vHTS is that screening millions of chemical compounds is computationally intensive: it has a high storage demand and is, therefore, termed as computational data challenge. Screening each compound, depending on structural complexity, can take from one to few minutes on a standard PC, which means screening a database with millions of chemical compounds can take years of computation time. Computational grid infrastructures are the best attempt to solving this problem thus far. The computing resources from EGEE grid infrastructure were utilized extensively [9-11], besides EGEE [12] several other grid infrastructures such as: AuverGrid [13], EELA [14], EUChinaGrid [15] and EUMedGrid [16] contributed significantly to the current project.

The combination of these two techniques (vHTS and Grid computing) will definitely decrease the financial and cost implications of rational drug design strategies. Several docking applications have already been run on grids and proved to be successful. Some of the success stories in *in silico *drug design on grid are the smallpox research grid [17], Anthrax research project and Cancer project [18-20].

WISDOM-I [21], the first large scale deployment of molecular docking application on EGEE, which took place from August 2005 to September 2005 has seen 42 million dockings, which is equivalent to 80 years of CPU. Virtual screening of 500,000 chemical compounds was performed by using FlexX against different plasmepsins (aspartic protease implicated in haemoglobin degradation). On the biological front three scaffolds were identified, one of them is guanidino scaffold, which is likely to be novel as they have not been reported as plasmepsin inhibitors before [22]. Experimental results have proved that the some of the compounds selected from WISDOM-I function as sub-micromolar inhibitors against plasmepsin [23].

The main goals of WISDOM project were to identify inhibitors to be tested in the experimental laboratories and to develop fault tolerant WISDOM production environment [24], which is capable of deploying molecular docking application efficiently on grid infrastructure. Drug targets from malaria are chosen initially, but this could be expanded to determine ligand binding to any target protein.

### The grid enabled virtual screening process

#### WISDOM-II, second large scale virtual screening on malaria

Previous work (WISDOM-I), has been focused on a single target family: the plasmepsin family of proteins. However, due to complexity of the life cycle and drug resistance more targets and more metabolic pathways have to be targeted to counteract the disease. Hence, in the current project: WISDOM-II, different validated targets involved in diverse metabolic activities of the parasite were selected. As different species of Plasmodium cause malaria to humans; in the current project, proteins not only from *Plasmodium falciparum*, but also from the *Plasmodium vivax *were embattled. The extension of the work to target *P. vivax *is due to its resurgence and casualties caused [25]. From Table 1, it is clear that targets were chosen as such to identify novel inhibitors for different proteins implicated in malarial life cycle with the idea in mind to interfere with resistance, consequently developing novel procedures and strategies for storage, post-processing, analysis of the docking results and finally selecting a representative set of potential inhibitors for further *in vitro and in vivo *testing.

**Table 1:** Structural features of the targets used in WISDOM II

Target	Activity	Structure	PDB id	Resolution Å	Cocrystallized Ligand	Co-factor
*Pf*GST	Detoxification	Dimer	1Q4J	2.2	GTX	NO
*Pf *DHFR (wild type)	DNA synthesis	Polymer	1J3I	2.33	WR99210	NADPH
*Pf *DHFR (Quadruple mutant)	DNA synthesis	Polymer	1J3K	2.10	WR99210	NADPH
*Pv*DHFR (wild type)	DNA synthesis	Polymer	2BL9	1.90	Pyrimethamine	NADPH
*P*vDHFR (Double mutant)	DNA synthesis	Polymer	2BLC	2.25	Des- chloropyrimethamine	NADPH

### Target structures

#### Glutathione-S-transferase

The *P. falciparum *glutathione S-transferase enzyme belongs to a super family of multifunctional, dimeric, phase II detoxification enzymes that can bind various xenobiotic, electrophilic substrates. Parasites as well as other rapidly dividing cells are highly dependent on a functional antioxidant defense system. For most parasites the sources of reactive oxygen species is mainly their high metabolic rate as well as oxidative stress imposed by the host's immune system. Additionally, the *P. falciparum *parasite performs haemoglobin degradation – a source of oxidative stress and free radicals [26]. The antioxidant defense system of *P. falciparum *is mediated by an ensemble of antioxidants like glutathione as well as antioxidant enzymes [27].

The primary function of GST lies in the protection of cellular macromolecules. GST deactivates harmful chemicals via the nucleophilic addition of the thiol (SH) group from glutathione (GSH), to the hydrophilic moiety of the toxic agent, thus rendering the electrophilic compounds harmless and enabling the removal of the substance. Because of the inactivation of potentially hazardous substances, GST activity is beneficial to an organism's health and survival [28,29]. In chloroquine-resistant parasites GST activity is directly and positively related to drug pressure [30,31].

Inhibition of GST will impair the general detoxification processes and, because the enzyme has peroxidase activity, reduce the antioxidant capacity of the parasite [32]. PfGST (EC 2.5.1.18) is a multi-functional protein consisting of two monomers. In accordance with other GST enzymes each monomer of PfGST contains an N-terminal α/β domain and C-terminal α-helical domain. The active site is defined by two binding sites: the G site, which binds GSH, and the more flexible H site, which can bind various other substrates. The monomers are predominantly held together by hydrophobic effects, but four salt bridges and four hydrogen-bonded pairs of residues also contribute to the dimerization [33,34]. The G site is relatively rigid and not greatly affected by inhibitor binding, with the exception of the C-terminal tail and the loop connecting the α-4 and α-5 helices. This region is very specific for its natural substrate (GSH). The recognition and binding occur via a network of polar interactions between PfGST and GSH.

The hydrophobic binding pocket (H site) is considerably more variable than the G site, due to the nature of second substrates. The substrate specificity of different isozymes in the GST super family can be attributed to the variation of amino acids present in the H site consequently leading to different interactions a ligand can form with amino acids in the H site of the enzymes [35].

PfGST also possesses a short μ-loop. In contrast to other μ-class GST enzymes, PfGST has only five residues after α-8, which is too short to form a wall or α-helix. This feature is lacking in PfGST, resulting in a more solvent-accessible H site. The result is that the H site is less shielded from solvents [35,36].

#### *Plasmodium vivax *and *P. falciparum *DHFR

*Plasmodium vivax *is becoming resistant to chloroquine and other antifolates, such as pyrimethamine [37-39]. The target enzyme of pyrimethamine is dihydrofolate reductase (DHFR). It was demonstrated that the resistance to pyrimethamine is caused by point mutation [40]. Interestingly, the crystal structure of DHFR enzyme from *P. vivax *was published by Kongsaeree *et al *in 2005 [41], where they indicated that the principal difference between DHFR wild type and mutant, implicated in the antifolate resistance, is a structural change in the chain of Asn-108, and this steric conflict is not present in *P. falciparum*.

Antifolates, such as pyrimethamine and cycloguanil, are the most exploited class of anti-malarials. To date, the most widely used antifolate is a combination of pyrimethamine, a dihydrofolate reductase (DHFR) inhibitor, and sulphadoxine, a dihydropteroate synthase (DHPS) inhibitor. DHFR and DHPS are two enzymes that belong to the folate biosynthetic pathway [42]. Although their synergistic action results in enhanced activity, their efficacy is seriously compromised by drug resistance. As a major advance towards the understanding of drug resistance in malaria, it has been demonstrated that drug resistance is due to single and multiple mutations of various amino acids in the DHFR and DHPS active sites in *P. vivax *as well as *P. falciparum *[43,44]. The analysis of the gene encoding *P. falciparum *DHFR from resistant parasites suggested that antifolate resistance arises from point mutations in the DHFR domain, mainly at positions 16, 51, 59, 108, and 164. It has been demonstrated that parasites with mutations at 16 and 108 have developed resistance to cycloguanil, with a thousand-fold drop in the K_i _compared with the wild type, whereas the K_i _of pyrimethamine is almost unaffected. On the contrary, there is cross-resistance between the drugs when multiple mutations at position 51, 59, 108 and 164 occur.

Combined homology modelling and molecular dynamics simulation studies proposed how pyrimethamine, cycloguanil and WR99210 (a third-generation antifolate) bind to wild type and resistant mutant *P. falciparum *and *P. vivax *DHFRs [45,46]. Crystal structure determination of the malarial DHFRs in complex with antifolates have confirmed and strengthened the proposed binding modes [46,47].

### Virtual docking procedure

The different steps of the virtual docking procedure will be described in the following section.

### Target preparation

The initial coordinates for all the target structures are obtained from Brookhaven protein database. Depending upon the inclusion of the significant residues, active site is defined as 8.0 – 10Ǻ around the co-crystallized ligands.

#### Glutathione-S-transferase (GST)

The X-ray crystal structure of GST utilized is 1Q4J [33]. 1Q4J is a homodimer with two chains A and B. The crystal water molecules are eliminated from the protein. The active site is defined as 8Ǻ around the co-crystallized ligand: GTX, all significant residues are included in the binding site. Re-docking with GTX ligand is performed for further optimization of the target parameters as well as software parameters.

#### *Plasmodium vivax *DHFR (PvDHFR) and *Plasmodium falciparum *DHFR (PfDHFR)

The protein structures used in this investigation are the crystal structures of wild-type *P. falciparum *DHFR (PDB code 1J3I) and of its N51I+C59R+S108N+I164L highly resistant mutant (PDB code 1J3K), both in complex with NADPH and the potent inhibitor WR99210, and the structures of wild type *P. vivax *DHFR (PDB code 2BL9) and of its S58R+S117N resistant mutant (PDB code 2BLC) in complex with pyrimethamine and des-chloro pyrimethamine, respectively [41,46]. The structures were cut at residue Asn231, which corresponds to the DHFR domain of the bifunctional DHFR-TS structure. Of the dimer, unit B was chosen because of its less missing residues. Met 1 was built as in unit A, and the position of missing residues from Asp87 to Asn90 was modelled with Modeller software [48]. At this purpose, the enzyme sequence with the four missing residues was aligned with the complete sequence, and ten models were generated with the Modeller software using 1J3I as template. The best model according to Prosa II was saved, and the coordinates of the four missing residues were inserted back in the original crystal structure. For the quadruple mutant of *P. falciparum *DHFR, the missing segment from residue 81 to 97 was taken from the wild type structure. *P. vivax *DHFR was prepared using the same methodology. Residues E24 and K48, which have truncated side chains in the original crystal structures, were assigned based on standard Amber topologies of amino acids. Residues from 84 to 105 missing in the double mutant structure were taken from the wild type structure.

All water molecules in the crystal structure were removed except for two conserved waters embedded into the protein (corresponding to W1249 and W1250 in the original 1J3I crystal structure) and close to the important residue D54. Hydrogens were added to the structures using the internal coordinates of the AMBER all-atom data base. All Lys and Arg residues were positively charged and Glu and Asp residues negatively charged. All calculations were performed with AMBER9 and the ff03 force field [49]. The parameters of the cofactor NADPH were taken from previous simulations [47,49].

The structures prepared as described above were refined with energy minimization, employing a distance-dependent dielectric constant e = 4r and a cutoff of 12 Å for non-bonded interactions. Firstly, 500 steps of conjugate gradient energy minimization were performed on the hydrogen atoms only, followed by 5,000 steps of minimization on the entire structure. Then, in order to refine the position of the hydrogen atoms added with Amber, 50 ps molecular dynamics at 300°K was performed on the hydrogens by adding strong restraints on the heavy atoms. Finally, 5000 steps of minimization were performed without restraints. All minimizations were performed on the protein structures with the corresponding antifolates bound in the active site (WR99210 or Pyr). For the antifolates, partial atomic charges on atoms were calculated with the AM1-BCC method [50] implemented in the antechAmber module of Amber9. Atom types and missing force-field parameters of the ligands were assigned based on the General Amber force-field (gaff) [51].

### Compound database

The compound library used for WISDOM was obtained from the ZINC database [52,53]. The ZINC database is a collection of 4.3 million chemical compounds from different vendors. ZINC library has been chosen because it is an open source database, and the data are available in different file formats (Sybyl mol2 format, sdf and smiles). So, basically, ZINC provides virtual compounds ready for virtual screening. A total of 4.3 million compounds were downloaded from the ZINC database and screened against targets mentioned in Table 1.

### FlexX: Docking software

The docking software used in the current study is FlexX [54,55]. It is extremely fast, robust and highly configurable computer program for predicting protein-ligand interactions. Standard parameter settings are used except for two cases: "Place particles" [56] and "Maximum overlap volume". These two parameters were subject to deliberate variation with FlexX. Since every target has a unique response to docking software parameters set, there is no generic solution to the parameter sets. Initial optimization experiments were done to arrive at a parameter set which gave best results in redocking experiment for a particular target.

### Setting up the platform before large-scale virtual screening

**Figure 1:**
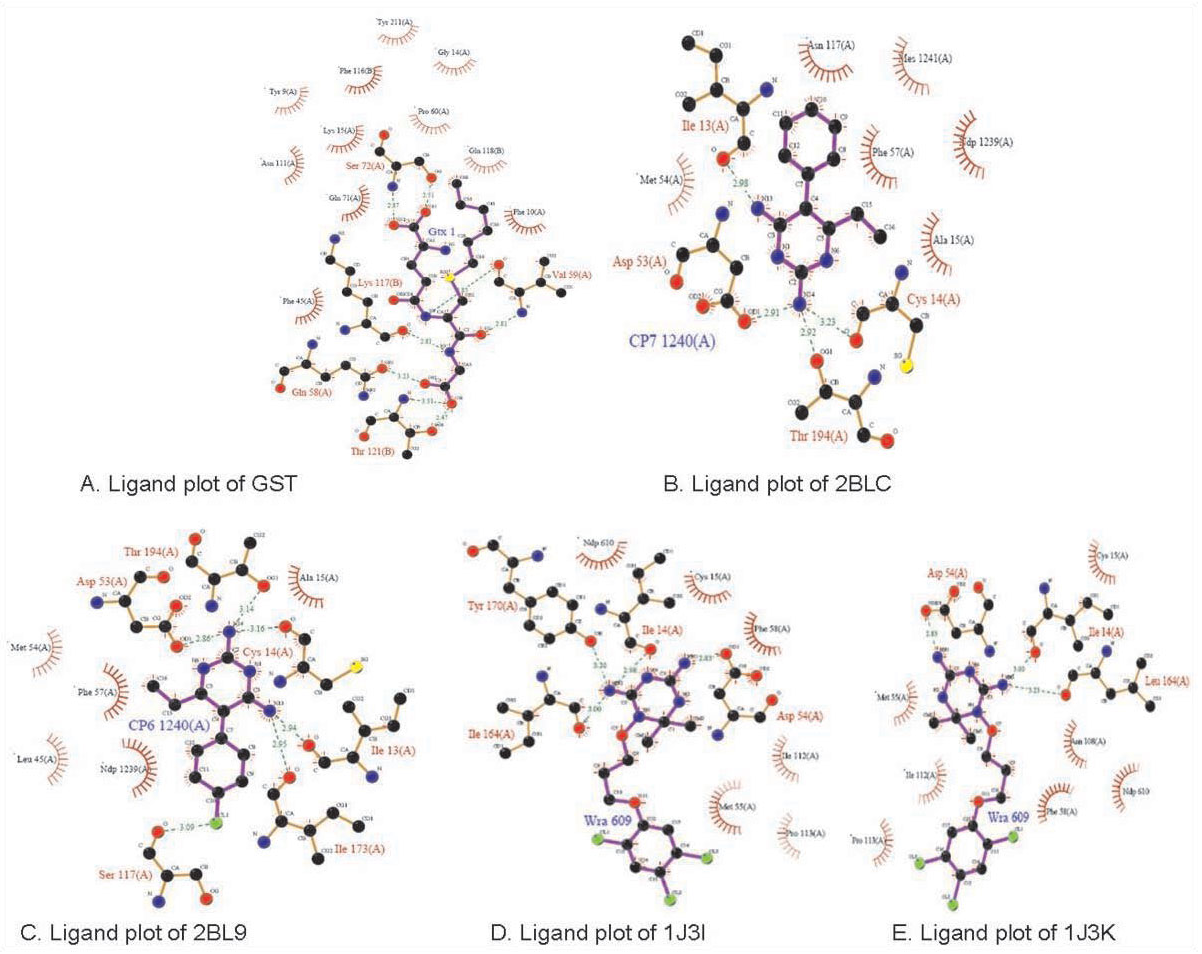
**Illustrates the ligand plots of targets used in the current study**. displays: A. Ligand plot of GST; B. Ligand lot of 2BLC; C. Ligand plot of 2Bl9; D. Ligand plot of 1J3I; E. Ligand plot of 1J3K. Ligand plots are obtained from Brookhaven protein database.

Re-docking can be defined as the removal of the co-crystallized compound (inhibitor or substrate) and then using a specific parameter set to dock this compound back into the active site of its target protein to validate the programs ability to dock novel compounds into the active site. These experiments serve as positive controls before large scale docking since aids in defining the active site and other simulation conditions. The docking pose during these experiments is validated by comparing the pose based on the RMSD between the atoms of the co-crystallized pose and the docking pose, as well as visual inspection of the orientation of the ligand. The lower the RMSD value and the more similar the docking pose to the co-crystallized ligand the better the docking results. Ligand plot information obtained from Brookhaven database serves as a template to validate the docking pose. The ligand plots of all the targets used in the current project are displayed in Figure 1. Ligand plots displays the binding mode of the co-crystallized ligand within the active site of the receptor, besides this it also describe the atom-to-atom interaction between the co-crystallized ligand to its respective receptor. This information is later compared with the docking poses before large-scale screening.

### Re-docking

**Figure 2:**
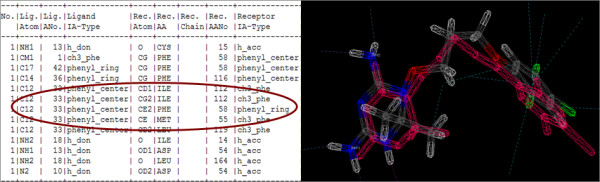
**Illustrates the redocking of WR9 ligand against 1J3K in parameter 8**. On the left hand side, Interaction information between ligand atom and target protein are displayed. On the right hand side redocked pose (CPK color) and reference coordinates (Red color) are displayed.

**Figure 3:**
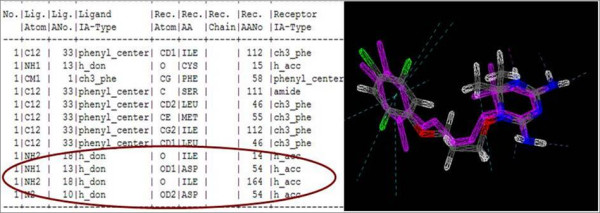
**Illustrates the re-docking of WR9 ligand against 1J3I in parameter 8**. On the left, Interaction information between ligand atom and target protein are displayed. On the right re-docked pose (CPK color: Corey, Pauling, Kultin color scheme) and reference coordinates (violet color) are displayed.

The results of the re-docking experiments are displayed in Table 2. Results are analysed at three levels: the RMSD (root mean square deviation) between the docking pose and the co-crystallized ligand, the docking score and the interaction information between protein and ligand. FlexX has a unique ability to compute the atom-to-atom interaction between the protein and the ligand. This information is exploited and further used in analysing the results. The docking poses of the co-crystallized ligands generated by FlexX are manually visualized and compared to their respective ligand plots. Two aspects were considered; the binding mode of the docking pose should be similar to ligand plot and should make interactions to the key residues of the receptor as described in ligand plots. Ligand plots are displayed in Figure 1. Table 2 displays the docking score and RMSD of the best docking conformation. For target PfGST (1Q4J: PDB ID), parameter set 1 performed better compared to other parameter sets. However 3.68 Å is still a big deviation (ideal RMSD should be <2 Å), but the binding mode the co-crystallized ligand adopted was quite convincing, as the docking pose was making interactions to the key residue. Besides that, the docking pose made interactions to the key residues responsible for the activity of the protein. In case of *P. vivax *DHFR (2BLC and 2BL9), the docking of the co-crystallized ligand did not perform well. The RMSD deviations were high (>4 Å) and the binding modes were not convincing. This is due to clashes between the protein and ligand atom surfaces. For PfDHFR re-docking is performed against protein structures before and after minimization by Amber software. Docking software parameters were tuned accordingly. For PfDHFR, we increased the maximum allowed overlap between the protein and ligand atom to diminish the van der Waals clashes. Re-docking against minimized structures with the same parameter sets gave best results. All the parameter sets reproduced the actual binding mode of the ligand, further made interactions to key amino acids and RMS deviation were less than 2 Å. Re-docking results of PfDHFR minimized structures are displayed in Table 3 and 4. Besides docking the co-crystallized ligand, well-known inhibitors against PfDHFR are docked. Table 3 and 5 displays the results of cycloguanil and pyrimethamine, WR9 under different docking parameter sets. Parameter 8 (maximum allowed overlap volume between protein and ligand surface: 100 Å^3^) gave the best results in terms of docking score, docking conformation and interactions to key amino acids. The results displayed in Table 4 correspond to the quadruple mutant results (1J3I: PDB ID) and Table 5 corresponds to the wild type results (1J3K: PDB ID). Figure 2 and 3 display the re-docking pose of WR9 against minimized structure of the Pf DHFR (1J3K) and Pf DHFR (1J3I), respectively, on the right hand side of the figure we can see the docking pose (CPK color) and reference co-ordinates in red color (IJ3K) and violet color (1J3I). On the left hand side protein ligand interactions are displayed. Highlighted are the interactions responsible for the activity of the protein (parameter sets 5, 6, 7, 8 correspond to maximum allowed overlap volume 10, 20, 30, 100 Å^3^respectively).

**Table 2:** Re-docking results of different targets in different parameter sets of FlexX

**Target**	**Ligand**	**Total Score**	**RMS-Value**	**Total Score**	**RMS-Value**	**Total Score**	**RMS- Value**	**Total Score**	**RMS-Value**
		**1**		**2**		**3**		**4**	
1Q4J_a	GTX	-24.33	3.68	-20.99	7.53	-20.15	6.94	-25.93	7.11
1Q4J_b	GTX	-19.93	6.45	-18.33	11.83	-18.33	11.78	-25.07	6.28
2BLC	CP7	-13.47	4.88	-14.09	4.78	-12.53	4.45	-14.43	4.78
2BL9	CP6	-13.657	4.71	-12.50	6.17	-13.65	4.71	-12.50	6.17
**Target**	**Ligand**	**Total Score**	**RMS-Value**	**Total Score**	**RMS-Value**	**Total Score**	**RMS- Value**	**Total Score**	**RMS-Value**
		**5**		**6**		**7**		**8**	
1J3K	WR9	-24.33	3.68	-23.91	1.81	-23.75	2.21	-26.41	3.14
1J3I	WR9	-30.75	2.49	-21.98	1.41	-20.83	2.69	-25.69	1.76

**Table 3:** Redocking results against quadrupule mutant DHFR

	**Best Score** **(kJ/mol)**	**RMSD for best solution** **(Å)**	**Rank for Best RMSD solution**	**Score for best RMSD** **Solution**	**Best RMSD** **(Å)**
QM_WR9_10	-23.22	3.37	106	-9.85	0.76
QM_WR9_20	-23.91	1.81	158	-9.85	0.76
QM_WR9_30	-23.75	2.21	97	-12.57	0.75
QM_WR9_100	-26.41	3.14	40	-18.06	1.14
QM_CYC_10	-23.25	1.46	525	-12.40	0.92
QM_CYC_20	-23.25	1.46	525	-12.40	0.92
QM_CYC_30	-23.25	1.46	146	-20.39	0.97
QM_CYC_100	-23.25	1.46	699	-15.08	1.01
QM_PYR_10	-23.68	1.21	8	-21.80	0.69
QM_PYR_20	-23.68	1.21	8	-21.80	0.69
QM_PYR_30	-23.60	1.26	16	-22.08	0.74
QM_PYR_100	-21.95	1.51	20	-20.31	0.97

QM = Quadruple mutant;

RMSD is in Angstroms;

Score is the free energy in kJ/mol

**Table 4:** Illustrates re-docking results against wild type DHFR

	**Best Score (kJ/mol)**	**RMSD for best solution** **(Å)**	**Rank for Best RMSD solution**	**Score for best RMSD** **Solution**	**Best RMSD** **(Å)**
WT_WR9_10	-30.75	2.49	57	-21.98	0.91
WT_WR9_20	-21.98	1.41	46	-13.78	0.91
WT_WR9_30	-20.83	2.69	2	-19.67	0.99
WT_WR9_100	-25.69	1.76	4	-22.47	0.83
WT_CYC_10	-24.36	1.43	622	-19.60	0.89
WT_CYC_20	-24.47	1.46	720	-19.88	0.95
WT_CYC_30	-24.47	1.46	7	-22.16	0.95
WT_CYC_100	-24.70	1.49	11	-31.49	0.97
WT_PYR_10	-29.72	1.25	6	-28.02	0.46
WT_PYR_20	-29.73	1.26	2	-27.70	0.53
WT_PYR_30	-29.73	1.26	2	-27.70	0.53
WT_PYR_100	-30.49	1.28	3	-30.31	0.49

WT = Wild type;

RMSD is in Angstroms;

Score is the free energy in kJ/mol

**Table 5:** Represents top compounds by docking against PfGST_a with interactions to key amino acids

**Compound**	**FlexX score**	**Interaction to Key AA'**
ZINC03989574	-50.586	10111
ZINC03989578	-49.698	11101
ZINC04847284	-49.698	11101
ZINC03930012	-48.396	10000
ZINC04522767	-47.633	11100
ZINC05808725	-47.006	01011
ZINC04068384	-46.956	01011
ZINC03948265	-46.286	11100
ZINC02748596	-46.117	11111
ZINC02102883	-46.016	01011

### Virtual screening on the EGEE grid infrastructure

After setting up the docking platform, virtual screening was performed on 4.3 million compounds against the targets specified in Table 1. Screening 4.3 million compounds on multiple target structures demands huge computation and storage resource power. EGEE and its related grid infrastructures (AuverGrid, EELA, EUChinaGrid and EUMedGrid) provided the resources required for executing the data challenge. Deployment strategies and WISDOM production environment were developed for submission of jobs and retrieval of results on the EGEE grid. More information on deployment and wisdom production environment can be found in Jacq *et al *[21]. The significant achievement on the grid side is the improved WISDOM production environment, which enabled smooth and successful deployment of docking jobs on the grid. The major difference between our previous deployments (WISDOM-I and the data challenge on avian flu) is the automatic resubmission of the docking jobs on the grid. Besides the enormous gain in the computing resources available, grid infrastructures enable also to store the tera bytes of scientific data and to share this data between the research laboratories located not only in different countries but also on different continents.

### Output data

The outputs of the docking results in FlexX are log files. All the results are stored and analysed by using MySQL databases. Three different forms of results are saved and analysed from each docking assay:

i. Docking scores of the ten best solutions after clustering

ii. Interaction information between protein and ligands of the ten best solutions,

iii. Binding modes of the ten best solutions.

Moreover, the ranking process is the integral part of the docking software. FlexX have a post processing optimization of the docking solution and clustering. Clustering in FlexX is based on RMSD, angle, and distance deviation (default values are used for the clustering; the necessity of clustering docking poses is discussed in [22]).

### Database schema to store the results

During the first deployments (WISDOM-I and data challenges on Avian Flu) the results were stored on the grid storage elements using the grid data management, this format made the analysis of the results particularly difficult, which left some room for improvement.

**Figure 4:**
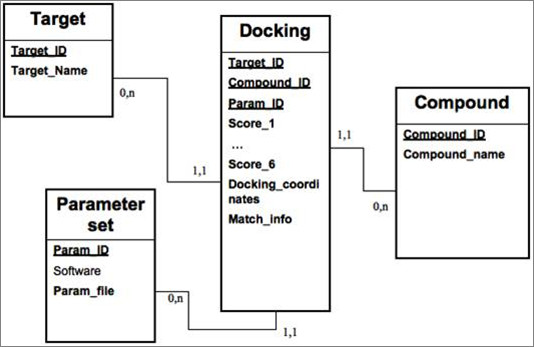
**A view of the result database schema used to store and analyse docking results**.

Since docking and scoring results often need to be extracted and parsed by biologists, user-friendly data retrieval systems need to be put in place. Hence, it was decided to rank the information based on the docking scores and do some initial filtering of the compounds. The relevant information was parsed directly into a relational database. The database was designed around the docking table (Figure 4), where docking scores are stored, which represent the binding free energy. The individual energy contributions to the total free energy of binding are also stored. This was useful for the filtering of compounds and it also helped in the docking results analysis. As shown in Figure 4, the insertion of records is performed directly at the end of the jobs. A simple perl script, using perl DBI library, parses the result file and builds, from the useful information, a query to insert the data from the grid to a remote MySQL server. The raw result files are also stored and replicated on the grid storage elements.

The real interest of such a solution is that the useful data are immediately available for query and analysis during the process. The usage of relational database along with SQL eases the selection of the best compounds as they can be selected accordingly to any attribute of the database tables. As almost all programming languages offer the ability to access database management systems through APIs, it will also ease the interoperability with web servers, for instance, if one wants to be able to monitor and view the data on a web interface.

### Strategies adopted for analysing the results

Result analysis is performed to identify a small number of promising compounds that can be tested further. During WISDOM-I, the compounds with best docking scores were visualized manually and interestingly observed that some of the top scoring compounds were making interactions to the key residues but the binding modes of the compounds were not optimal and not comparable with docking poses of co-crystallized ligands, one of the reason may be due to the rigid nature of the receptor. Consequently, the result analysis took place by first extracting the compounds based on docking scores and then by rescoring the best docked ligands with more sophisticated scoring functions. At this purpose, for this project, an automated refinement and rescoring procedure developed by Rastelli *et al *[57], which refines ligand-target complexes with molecular mechanics and molecular dynamics, and then calculates the binding free energies according to MM-PBSA and MM-GBSA methods are utilized. Such procedure, called BEAR (Binding Estimation After Refinement) [58] significantly improved the overall procedure and resulted in the identification of plasmepsin inhibitors (WISDOM-I). Hence, the same procedure was used to extract the best 5,000 (against GST), 15,000 (against DHFR) compounds based on docking scoring values. MM-PBSA and MM-GBSA (Molecular mechanics-Poisson Boltzmann surface area and Molecular Mechanics-Generalized Born surface area) procedures calculate the absolute free energies for non-covalent interactions of protein and ligand in solution by using force field based molecular mechanics method [59,60].

**Figure 5:**
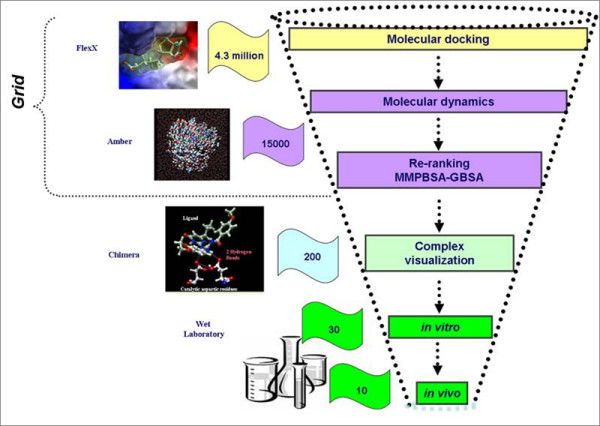
**Overall filtering process employed in WISDOM-II project**. demonstrates overall filtering process employed in WISDOM-II project. The first three steps in the workflow (Docking, Molecular dynamics and rescoring by MMPBSA, MM-GBSA) are performed on Computational grids and the visualizations by chimera software are performed manually on the local machine of the user.

The common filtering process employed in WISDOM project is displayed in Figure 5. After rescoring by molecular dynamics methods, the compounds are further manually visualized by using Chimera software [61] and other structural visualizing software. Interactions to key residues of the receptor and binding mode of the ligand are main criteria for further selecting the compounds to be tested in experimental laboratories.

## Results and discussion

### Docking results

Docking results of PfGST are represented in Table 5. Six amino acids were considered to be responsible for the activity of the target: Tyr9, Gln58, Val59, Ser72, Gln71 and Lys15. Chemical compounds interacting with these amino acids were of significance and hence computed. All ten top scoring compounds displayed in Table 5 made interactions to these key amino acids. A binary scoring mode was adopted for the residue reactions in Table 5, column 3: "0" represents false (no interaction with the specified amino acid) and "1" represents true (either a hydrogen bond or a hydrophobic interaction, was made). From Table 5, it is clear that all the top scoring compounds are making interactions with at least one of the key amino acids. These observations are later compared to the standard protein ligand interaction information obtained from ligand plots displayed in Figure 1. This particular method not only allowed us to select compounds based on scoring but also based on interaction information (hydrophobic and hydrophilic interactions), which is very significant from the structural point of view for the identification of hits.

### Diversity analysis of top scoring compounds for PfGST and PfDHFR

**Figure 6:**
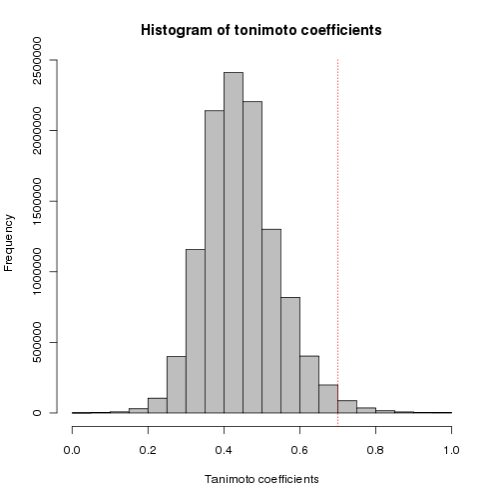
**Diversity analysis of the top scoring 5000 compounds against PfGST**. Demonstrates diversity analysis of the top scoring 5000 compounds against PfGST. The red line on the histogram is placed at TC value 0.7 and large bars on the left hand side before the red line indicates, the compound dataset is diverse.

**Figure 7:**
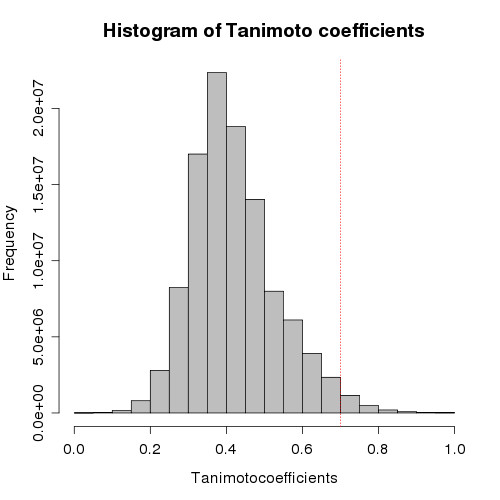
**Diversity analysis of the top scoring 15000 compounds against PfDHFR**. Demonstrates diversity analysis of the top scoring 15000 compounds against PfDHFR. The red line on the histogram is placed at TC value 0.7 and large bars on the left hand side before the red line indicates, the compound dataset is diverse.

To give wide overview on the results obtained by docking, diversity analysis against the PfGST best 5,000 compounds and PfDHFR best 15,000 compounds by docking score was performed by using MOE software [62]. Finger prints of all the compounds were created by using FP: BIT MACCS and then used Tanimoto coefficient (TC) for calculating the diversity among the compounds [63]. At similarity cut off of Tanimoto coefficient 0.7, out of 5,000 compounds of PfGST, 3,394 different clusters were identified by this method, which indicates the best 5,000 compounds diverse and dissimilar. The Tanimoto coefficient (similarity = the number of bits set in both molecules divided by the number of bits set in either molecule) is a validated and most commonly used similarity coefficient in chemical informatics while calculating diversity of the chemical compound database. It ranges from values 0 to 1, while value "1" corresponds to completely similar compound and "0" completely disssimilar). Diversity analysis is performed to demonstrate the best compounds by docking score are diverse enough for further analysis and for the identification of novel scaffolds. Pair wise frequency (Y-axis) and Tanimoto coefficient value (X-axis) are plotted and displayed in Figure 6. The values of mean, median, 1^st ^quartile, 3^rd ^quartile of the histogram are 0.44, 0.43, 0.37, 0.50 respectively. The 1^st ^quartile and 3^rd ^quartile values signify that 25% of the compounds possess TC values of 0.37 and 75% of the compounds possess TC values of 0.5. For PfDHFR diversity analysis is performed against 15,000 top scoring compounds. Pair wise frequency (Y-axis) and Tanimoto coefficient value (TC) (X-axis) are plotted and displayed in Figure 7. The values of mean, median, 1^st ^quartile, 3^rd ^quartile of the histogram are 0.42, 0.40, 0.34, 0.48 respectively. The 1^st ^quartile and 3^rd ^quartile values signify that 25% of the compounds possess TC values of 0.34 and 75% of the compounds possess TC values of 0.48. These observations and figures indicate that the top scoring compounds are diverse and have potential to find novel compounds. The frequency on the Y-axis represents pair wise similarity of each compound against all the compounds in the database (5,000 × 5,000 times for PfGST).

### Modeling aspects of final hits against PfGST

**Figure 8:**
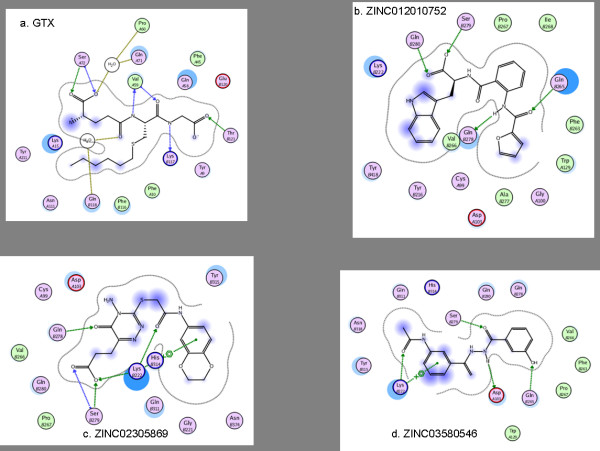
**PfGST-compound hydrogen bonding interaction**. Displays PfGST-compound hydrogen bonding interaction. Interaction informations are displayed for the best compounds which have comparable hydrogen bonding pattern like that of co-crystallized ligand, a.GTX (See table 6 for summary of interactions for best compounds).

To understand the interactions between PfGST and final hits, the ligand plots for each complex (PfGST and the compound) were generated and further visualized manually. Protein ligand interactions are studied in three dimensions and for clarity in displaying they are depicted as 2D interaction diagrams. These interactions presented here are generated using the ligand plot module of MOE software. It is evident from Figure 8 that inhibitors are located in the center of the active site, and are stabilized by hydrogen bonding interactions. The hydrogen bonding information along with their distances is listed in Table 6. Figure 8 displays the binding modes of the five best compounds in the active site of the PfGST_a chain. To allow the comparison of binding mode of the compounds and co-crystallized ligand, ligand plot and interactions information is generated for GTX (Cocrystallized ligand of PfGST). It is obvious from Table 6 and Figure 8 that the compounds listed here possess comparable binding poses and patterns. Especially compounds ZINC03533756, ZINC03830430, ZINC03580546, ZINC02305869 generated interaction patterns very similar to the one observed with GTX; making hydrogen bonding to Val59 and Ser72 with backbone as well as with side chains of the amino acids.

**Table 6:** PfGST interactions against best compounds are displayed

***Ligand Name***	**Ligand---Protein**	**Protein Residue**	**Type of interaction**	**Distance Ǻ**
GTX	1. N--O & O---N	1. Val59	1. H-don & H-acc	1: 2.85 & 2.81
	2. O---OG & O---N	2. SER72	2. H-acc & H-acc	2: 2.51 & 2.87
	3. N---O	3. LYS117	3. H-don	3: 2.81
ZINC012010752	1. N---OE & O---NE	1. GLN71	1. H-don & H-acc	1: 1.93 & 3.02
	2. O---OG	2. SER72	2. H-acc	2: 2.78
	3. O---NE	3. GLN56	3. H-acc	3: 3.02
ZINC01788367	1. O---N & O---OG	1. SER72	1. H-acc & H-acc	1: 3.02 & 2.89
				
ZINC02305869	1. O---NZ & O---NZ	1. LYS15	1. H-acc & H-acc	1. 2.97 & 2.93
	2. O--NE	2. GLN71	2. H-acc	2: 2.99
	3. O---N & O---OG	3. SER72	3. H-acc & H-acc	3: 2.89 & 2.83
ZINC02449312	1. O---OG	1. SER72	1. H-acc	1: 2.89
ZINC03533756	1. N--O & O---N	1. Val59	1. H-don & H-acc	1: 2.11 & 3.05
	2. O---OG & O---N	2. SER72	2. H-acc & H-acc	2: 3.04 & 2.92
ZINC03580546	1. N---OD	1. ASP105 (B)	1. H-don	1: 2.30
	2. O--NE	2. GLN58	2. H-acc	2: 3.11
	3. O---OG	3. SER72	3. H-acc	3: 2.99
ZINC03830430	1. O---N	1. Val59	1. H-acc	1: 2.91
	2. O---N	2. SER72	2. H-acc	2: 2.92
ZINC05225308	1. O---NZ & O---NZ	1. LYS15	1. H-acc & H-acc	1: 2.95 & 3.28
	2. O--NE	2. GLN71	2. H-acc	2: 2.92
	3. O---N & O---OG	3. SER72	3. H-acc & H-acc	3: 3.30 & 2.92
ZINC02453649	1. O---NE	1. GLN56	1. H-acc	1: 2.93
	2. O---N & O---OG	2. SER72	2. H-acc &. H-acc	2: 2.92 & 2.82

Especially displays H-bond interactions. In the table, column 2 represents the ligand atom to protein atom interaction, column 3 represents the protein residue against which the compound made the interaction, column 4 represents the type of interaction, column 5 represents the distance at with the H-Bond is formed.

## Conclusion and prospective

In this paper, the potential impact of grid infrastructures for in silico drug discovery is demonstrated. The effort described here focussed on two malaria biological targets, DHFR and GST, but at much reduced cost, the same strategy can be applied to produce focused compound libraries for any other malaria targets. Through this article, the intention is to draw the attention of the research communities working on these neglected diseases to the opportunity offered by this grid-enabled virtual screening approach for producing short lists of particularly promising molecules, which can be tested in vitro at a reduced cost. Besides the use of the computational grids for producing large amount of scientific data, grids form a platform for the convenient global exchange of the chemical data produced.

One major bottleneck in large scale screening experiments is the handling of large data output of these experiments. As shown in this paper, this problem is addressed by parsing the results into a MySQL database, storing the docking score as well as atom-to-atom interaction between the protein and ligand. The interaction information plays a vital role in selecting the hits, since it takes the compound counterpart, the protein, into consideration as well.

Diversity analysis (using finger prints, MACCS keys and Tanimoto Coefficient) was performed on the best compounds based on docking results and revealed that the compounds are quite diverse and sensible for further analysis. Future works aims at two things: an extension of the virtual screening pipeline by additional analysis methods and an even tighter integration of in silico prediction of candidate molecules and experimental validation of the compounds.

As an extension of the in silico pipeline for virtual screening, data handling and data analysis methods have to be improved significantly. The storage of docking results in the database was just a first step; in the future it is expected to be able to learn from in silico experiments by analysing entire series of docking experiments. Techniques supporting the judicious selection of chemical compounds from the large scale screening data will need to be improved. New features of drug-like molecules such as their potential toxicity will have to be addressed by an extension of the in silico screening through predictive toxicology systems. On the long run, it is likely to extend the in silico drug discovery workflow by models for predictive ADME. The rather proprietary nature of the drug discovery process in the pharmaceutical industry resulted in limited availability of models in this field, but initiatives such as the European Innovative Medicine Initiative (IMI) might help to foster broader uptake of computational models for predictive ADME (and toxicity) by altruistic research initiatives, such as WISDOM.

The current study may serve as a template for finding hits cost effectively by utilizing the *in silico *methods against multiple targets at the same time. The WISDOM collaboration is also keen to receive requests for docking other malarial targets according to the procedure described in this paper.

## Competing interests

The authors declare that they have no competing interests.

## Authors' contributions

VK wrote the manuscript. VK and JS performed the submission of jobs on the Grid infrastructure. VB and MHA conceived the project, obtained funding and supervised the study. AD, MB, VK performed data extraction, formatting and analysis for PfGST. GR performed the modeling studies for DHFR target before the large scale screening.
